# Influenza-related severe acute respiratory infection in the north of Vietnam: healthcare burden and economic impact

**DOI:** 10.1186/2047-2994-4-S1-P14

**Published:** 2015-06-16

**Authors:** YTT Nguyen, TB Nguyen, TP Nguyen, TH Nguyen, HH Vu, MTQ Le, DN Tran, TT Do, JM Partridge, JC Kile, TV Nguyen, HT Nguyen

**Affiliations:** 1 National Institute of Hygiene and Epidemiology, Hanoi, Viet Nam; 2 Program, U.S. Centers for Disease Control and Prevention, Hanoi, Viet Nam; 3 Bill and Melinda Gates Foundation, Seatles, USA; 4 Provincial Preventive Medicine Center, Thai Binh, Viet Nam

## Introduction

The disease and economic impact of seasonal influenza viruses is not well described for severe acute respiratory infection (SARI) patients in Vietnam.

## Objectives

To describe the morbidity, mortality, and economic impact of SARI at hospitals in Thai Binh province in Vietnam.

## Methods

A modified WHO's SARI definition (temperature ≥38^0^C, AND cough or sore throat, AND shortness of breath or difficulty in breathing, AND hospitalization) used to enroll cases in three hospitals. A standardized questionnaire used to collect data on the direct and indirect costs of hospitalization and treatment, including patient work days and care-giver days lost. A throat swab was collected for influenza virus detection by RT-PCR.

## Results

There were 7,833 SARI cases in 2013. Of 1,295 (17%) SARI cases tested, 229 (18%) were positive for influenza viruses, of which 59% were male. The proportion of influenza positives by age group was 69% in 0 to <5 years, 10% in 5 to <15, 4.5% in 15 to <50, 3.6% in 50 to <65, and 13% in ≥ 65. Influenza viruses identified were A/H1N1pdm09 (41.2%), A/H3N2 (30.3%), B (26.7%), co-infection of A/H3 & B and A/H1N1pdm09 & B (1.8%). Among all SARI patients, there were 12 (0.15%) deaths, of which 2 (16.7%) were positive for influenza B. Of 1,295 cases, 3 (<1%) reported receiving an influenza vaccine during the previous 12 months. For influenza-positive SARI cases, the median hospital stay was 8 days (IQR 5–8). Economic impact included direct and indirect costs per patient of US $176, an average of 7 work days lost for the patient and 10 care-giver days lost. The household monthly income of all SARI cases surveyed is US $58, (income in Thai Binh province in 2013 estimated US $100 per person per month).

## Conclusion

Influenza-related SARI is a burden to healthcare in Thai Binh province and has an economic impact on patients. The average total cost for 8 days of treatment is approximately 3 times the household monthly income. The rate of influenza vaccination was very low. The burden of SARI relative to the influenza and other respiratory viruses in Vietnam need to be studied further.

## Disclosure of interest

None declared.

